# Wavefront analysis and phase correctors design using *SHADOW*

**DOI:** 10.1107/S1600577524002728

**Published:** 2024-04-23

**Authors:** Hossein Khosroabadi, David Laundy, Vishal Dhamgaye, Kawal Sawhney

**Affiliations:** a Diamond Light Source, Harwell Science and Innovation Campus, Didcot OX11 0DE, United Kingdom; IOM-CNR and Elettra-Sincrotrone, Italy

**Keywords:** X-ray optics, wavefront error, ray-tracing simulation, refractive wavefront corrector, knife-edge imaging

## Abstract

Knife-edge imaging is a successful method for determining the wavefront distortion of focusing optics such as Kirkpatrick–Baez mirrors or compound refractive lenses. In this study, the wavefront error of an imperfect elliptical mirror is predicted by developing a knife-edge program using the *SHADOW*/*OASYS* platform. It is shown that the focusing optics can be aligned perfectly by minimizing the parabolic and cubic coefficients of the wavefront error. The residual wavefront error provides precise information about the figure/height errors of the focusing optics suggesting it as an accurate method for *in situ* optical metrology. A Python program is developed to design a customized wavefront refractive corrector to minimize the residual wavefront error. Uniform beam at and out of focus and higher peak intensity are achieved by the wavefront correction in comparison with ideal focusing. The developed code provides a quick way for wavefront error analysis and corrector design for non-ideal optics especially for the new-generation diffraction-limited sources, and saves considerable experimental time and effort.

## Abstract 2

A quick way of wavefront error analysis of a focusing optics using *SHADOW* is presented, that is required for fourth-generation light sources. The method is developed to optimize the focal spot and intensity of a non-ideal optics by designing a customized phase corrector.

## 1. Introduction

Fourth-generation synchrotron facility upgrades and free-electron lasers have in recent years aimed to enhance the beam brightness and coherence to considerably higher levels than the present third-generation facilities. Higher flux, smaller focal size toward the diffraction limit and a variable-size uniform profile are among the important requirements for many experimental techniques such as diffraction, macromolecular crystallography and spectromicroscopy at these facilities. To explore these advantages, especially close to the diffraction limit, a nearly ideal focusing optics and its perfect alignment are needed. Therefore, investigating the wavefront error caused by optics misalignment and imperfections such as figure/height error and its correction have become more and more popular in recent years (Laundy *et al.*, 2019 ▸; Seiboth *et al.*, 2017 ▸; Schropp *et al.*, 2013 ▸). Various experimental techniques such as speckle (Bérujon *et al.*, 2012 ▸, 2013 ▸, 2020 ▸), ptychography (Lyubomirskiy *et al.*, 2019 ▸; Moxham *et al.*, 2020 ▸, 2021 ▸; Schropp *et al.*, 2013 ▸), grating interferometry (Weitkamp *et al.*, 2005 ▸; Ziegler *et al.*, 2007 ▸; Diaz *et al.*, 2010 ▸), Hartman sensors (Idir *et al.*, 2010 ▸) and knife-edge imaging (Liu *et al.*, 2020 ▸; Laundy *et al.*, 2019 ▸) have been introduced to measure the wavefront error. Each of them has its advantages and drawbacks. Various methods have been developed to reduce the wavefront error and achieve a diffraction-limited spot by the available optics (Laundy *et al.*, 2019 ▸; Seiboth *et al.*, 2017 ▸; Mimura *et al.*, 2010 ▸). Among them, our team at Diamond Light Source have developed a knife-edge imaging technique (Laundy *et al.*, 2019 ▸) to measure the wavefront error and used refractive correctors (Laundy *et al.*, 2019 ▸; Sawhney *et al.*, 2016 ▸) to minimize it.

The knife-edge wavefront sensing method measures the intensity from a set of images acquired by an area detector while a knife-edge is scanned through the focal spot. The technique has been successfully applied to different focusing optics such as elliptical mirrors, the Kirkpatrick–Baez (KB) mirror system (Laundy *et al.*, 2019 ▸), compound refractive lenses (Dhamgaye *et al.*, 2020 ▸; Sawhney *et al.*, 2019 ▸) and variable focusing lenses (Dhamgaye *et al.*, 2023 ▸). The correction of wavefront error has been demonstrated by customized (Sawhney *et al.*, 2016 ▸) or adaptive (Laundy *et al.*, 2019 ▸) refractive structures made from SU-8 polymer fabricated by deep X-ray lithography at the Indus-2 synchrotron (Dhamgaye *et al.*, 2014 ▸). The customized structure is specifically designed for a known height error profile and energy while the adaptive one can correct low harmonic wavefront error for different optics and over a wider photon energy range using two in-line sinusoidal profile correctors (Laundy *et al.*, 2019 ▸). In both cases, considerable time and effort are needed to collect a series of experimental data and to optimize the wavefront correction. In this study, we present a simulation method that facilitates a short turnaround by determining the wavefront error profile for an X-ray optic, designing the suitable corrector and predicting the level of wavefront correction. This simulation method is demonstrated here for an elliptical mirror, and it can be extended to a variety of X-ray optics including some complex optical schemes such as two-dimensional wavefront correction for KB mirrors, combined reflective–refractive optics, highly curved optics and multilayer optics.

The available beamline design and X-ray optics simulation codes are mainly divided into two categories: geometrical ray-tracing such as *SHADOW* (Sanchez del Rio *et al.*, 1992 ▸; Rebuffi & Sanchez del Rio, 2016 ▸) and *RAY* (Schafers, 2008 ▸), and wavefront propagation such as *SRW* (Chubar & Elleaume, 1998 ▸; Chubar *et al.*, 2013 ▸) and *PHASE* (Bahrdt, 1997 ▸; Bahrdt & Flechsig, 1997 ▸). *SHADOW* has been widely used at synchrotron radiation facilities during the last decades (Sanchez del Rio & Rebuffi, 2023 ▸) to design X-ray beamlines where the coherency and diffraction effects are less involved. *SRW* instead propagates the beam by considering phase effects, so it is more appropriate for diffraction-limited fourth-generation light sources or coherent X-ray free-electron laser sources (Chubar *et al.*, 2023 ▸). The considerably higher computational cost and fewer optical element choices in *SRW* compared with *SHADOW* mean a compromise must be made when selecting which code is the best to use. Intermediate solutions, however, are required for partially coherent sources to balance the computational cost and complexity of the wavefront propagation with the phase diffraction requirements. For example, the hybrid method, a combination of *SHADOW* ray-tracing with wave propagation (Shi *et al.*, 2014 ▸), has been able to simulate diffraction effects due to slit edges or mirror height error. We have developed an alternative simulation approach using a knife-edge technique that takes into account the diffraction effects by wavefront error analysis. Both studies consider the diffraction effects from ray-traced data using *SHADOW*. The present study, however, develops the method further to design the refractive wavefront corrector and to predict the height error profile by an *in situ* metrology.

In this study, wavefront analysis is carried out using our developed knife-edge simulation method in the *SHADOW* ray-tracing software and comparing with earlier published experimental results (Laundy *et al.*, 2019 ▸). The method is further established for designing a customized wavefront corrector for an elliptical mirror and estimation of the level of wavefront correction by use of a simulated wavefront corrector.

## 2. Method

**Figure 1:**
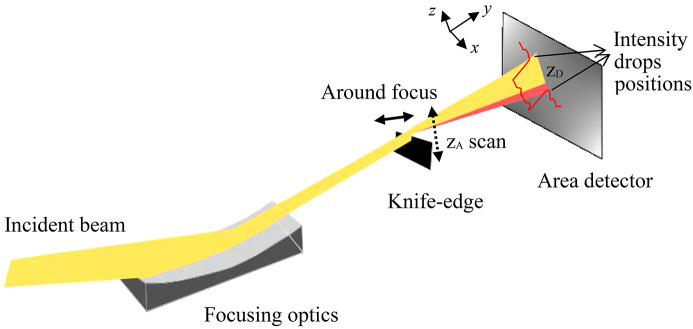
Schematic layout of the knife-edge imaging method for a one-dimensional focusing optic. While the yellow intensity passing above the knife-edge is expected, the red part is coming from the wavefront error. The red profile illustrates a schematic intensity histogram on the detector in the vertical direction. The reference frame is defined as: *y* along the beam direction, *x* and *z* in the sagittal and tangential directions, respectively, on the optical element and horizontal and vertical on the slits, knife-edge and detector.

The knife-edge imaging method, schematically shown in Fig. 1 ▸, is a fast and accurate technique for measuring the wavefront error profile of a focusing X-ray optic. A sharp-edge blade made of an absorbing material such as gold (knife-edge) is scanned through the beam spot, and the intensity projected onto a downstream surface area detector is measured at each knife-edge position. The measured intensity profile is analysed to find a step-like drop at each pixel of the detector for a knife-edge position. As shown in Fig. 1 ▸, the beam deviation from the focal position due to the wavefront error induces a shift in the step position. The rays are traced back geometrically to the focusing optics to determine the slope of the wavefront error. The wavefront sensing by knife-edge technique is based on the geometrical deflection of the rays at the plane of the knife-edge, so *SHADOW* can treat them in a similar way. The wavefront calculated in *SHADOW* is then applied as a phase difference for a fully coherent source to propagate the rays to the focus or out-of-focus positions. The Loop Function widget in the *OASYS* user interface (Sanchez del Rio & Rebuffi, 2019 ▸) allows easy and quick calculation of the knife-edge scans and intensity histogram collection.

**Figure 2:**
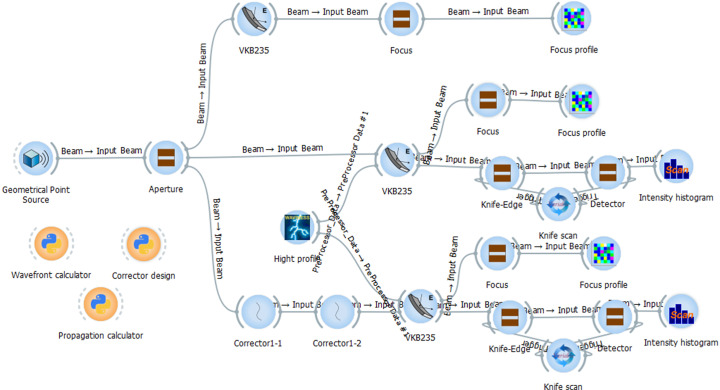
*OASYS* workspace used to develop the knife-edge simulation method to determine the wavefront error profile and design of the wavefront corrector profile. Top, central and bottom *OASYS* widget paths are prepared for the wavefront analysis of the ideal mirror, surface height deformed mirror, and wavefront correction of the deformed mirror by the refractive corrector, respectively. Macros widgets show the Python scripts to calculate the wavefront, design of the corrector and wave propagation.

*SHADOW* in the *OASYS* interface (Sanchez del Rio & Rebuffi, 2019 ▸) has been used for the ray-tracing calculation of the optical layout. The prepared *OASYS* workspace with widgets is shown in Fig. 2 ▸. A geometrical point source is used to simplify the simulation. An elliptical mirror with a demagnification of 180 as a vertical focusing mirror (VFM) is placed at 42.5 m from the source. The incident beam to the mirror is apertured by a 270 µm × 270 µm square aperture at the mirror position.

The height error profile for the mirror is produced by the Waviness widget in *OASYS*. The knife is simulated by an obstructed slit with zero transmissivity which is scanned along the transverse direction. The intensity histogram on the detector, 0.5 m downstream of the slit widget, is recorded for each position of the knife by the Loop Point widget. A Python program is developed to calculate the wavefront by analysing the recorded histogram intensities. The refractive corrector is defined as a double flat widget, one for each face, at zero distance from each other. The thickness profile of the corrector is calculated by a separate macro and fed into the corrector widget by the surface error option. The optical constants of the SU-8 based corrector structure are calculated by *XOP* (Sanchez del Rio & Dejus, 2011 ▸). An iterative loop, if required, is followed to determine the shape and profile of the wavefront corrector structure that provides the lowest root-mean-square (RMS) wavefront error. Finally, the propagation of the beam around the focus, for uncorrected and corrected wavefront error of the mirror, is calculated by Fresnel–Kirchhoff propagation, which is explained elsewhere (Born & Wolf, 2001 ▸).

## 3. Results

### 3.1. Wavefront error analysis

**Figure 3:**
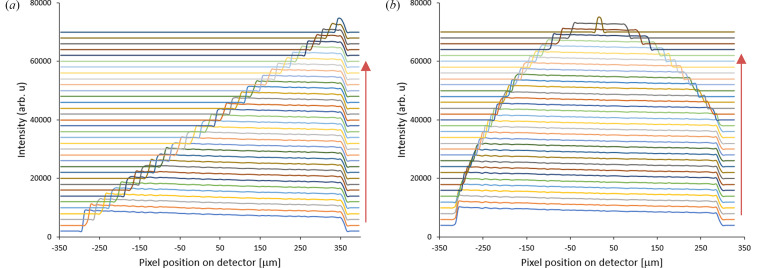
Typical vertical intensity histograms on the detector for different values of the vertical position of the knife. Histograms are plotted with a vertical shift, and the red vertical arrows show the knife-edge position from before intersecting the beam (the lowest histogram) to fully blocking the beam (the highest histogram). The knife-edge relative position to the focus along the beam and mirror pitch offset are, respectively, +1.0 mm (downstream) and 0 µrad (*a*) and −3.6 mm (upstream) and −50 µrad (*b*). The intensity drops remain in a vertical line for ideal focusing.

Fig. 3 ▸ shows a typical series of intensity histogram profiles along the vertical direction on the detector (*z*_D_) for different knife-edge positions along the beam direction, *y*, and VFM grazing incident (pitch) angles. Then, *z*_D_ is traced back to the mirror aperture to find the position *z*_A_ on the aperture. The intensity drop position on the aperture, *u*(*z*_A_), changes by the knife-edge position and can be expanded to the first few orders for small aperture (paraxial regime) as

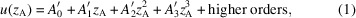

where 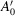 to 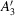 are the coefficients from the zeroth to the third order. As the derivative curve reflects the angular distortion of the wavefront, it can also be derived from the beam phase space *z*–*z*′ in the vertical direction. Equation (1) can then be integrated to find the wavefront error, *w*(*z*_A_), as

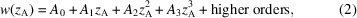

where *A*_0_ and *A*_1_ correspond to a constant phase shift and a tilt of the radiation field, while *A*_2_ = 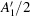 (parabolic) and *A*_3_ = 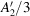 (cubic) coefficients indicate the level of the focusing optics misalignment, respectively, for the focal position and the pitch angle (Laundy *et al.*, 2019 ▸).

**Figure 4:**
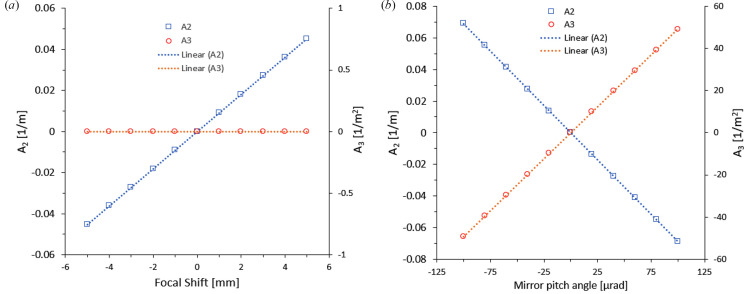
Cubic (*A*_3_) and parabolic (*A*_2_) coefficients of the wavefront error are calculated for the focal shift position at 3 mrad fixed pitch angle (*a*) and for a mirror pitch angle shift at the original focal position (*b*).

The *A*_2_ and *A*_3_ terms are plotted in Fig. 4 ▸ for several longitudinal knife-edge positions and VFM pitch angles. It is noted that both *A*_2_ and *A*_3_ behave linearly in this shift range, and the slopes are constant for a specific mirror. The data are in good agreement with the coefficients that have been obtained experimentally for this mirror at the B16 Test beamline (Sawhney *et al.*, 2010 ▸). The focal position misalignment only changes the parabolic coefficient, while the pitch misalignment changes both parabolic and cubic coefficients. So, these calibration curves can be used for accurate alignment of a focusing mirror after obtaining *A*_2_ and *A*_3_. Experimentally, the cubic and parabolic terms are normally minimized by tweaking the mirror pitch angle and the knife-edge position in several iterations. The simulation helps to predict both values in one run by having starting values of cubic and parabolic terms. A two-dimensional scan is needed for a KB system or toroidal mirror that focuses the beam in both vertical and horizontal directions. However, in most cases the sagittal direction can be ignored due to the forgiveness factor (Castro & Reininger, 1991 ▸; DiGennaro *et al.*, 1988 ▸). The height error in the sagittal direction can be ignored and each mirror in a KB pair can be deconvoluted to only tangential consideration due to this factor.

**Figure 5:**
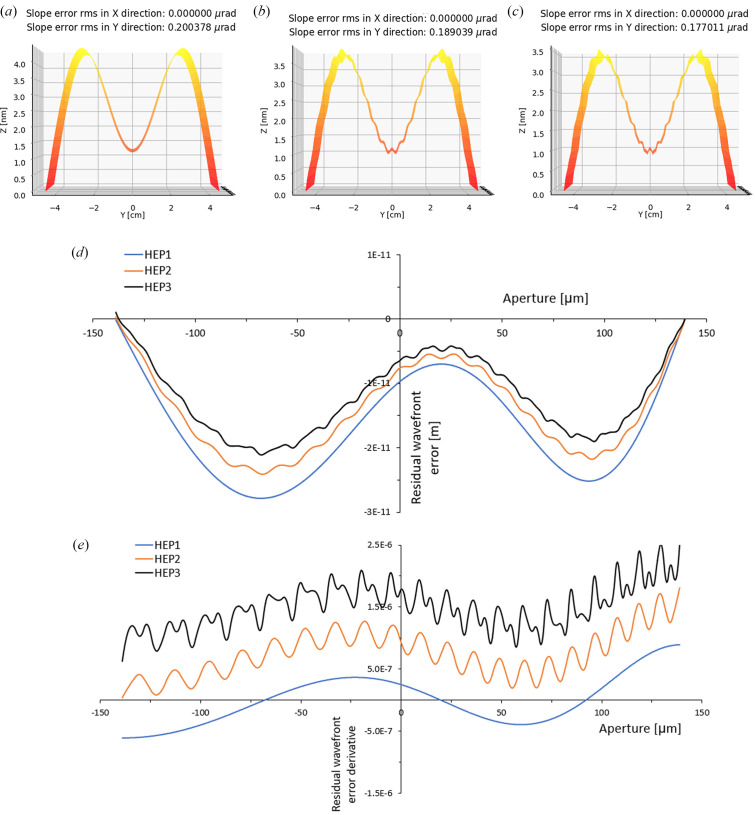
Typical height error profiles on the mirror including 0th and 1st harmonics (HEP1) (*a*), 0th, 1st and 20th harmonics (HEP2) (*b*), and 0th, 1st, 20th and 40th harmonics (HEP3) (*c*) produced by the waviness widget in *OASYS*. The corresponding residual wavefront error (*d*) and residual wavefront error derivative (*e*) are calculated by the model described in Section 2 and plotted by a relative vertical shift.

The mirror height error (HE) produces other contributions to the wavefront error in addition to the polynomial functions described in equation (2). This is called the residual wavefront error and can be expanded by a series of sin/cos terms instead, similar to the approach used for mirror height error analysis by Sanchez del Rio & Marcelli (1992 ▸). To investigate the residual wavefront error, three typical height error profiles are produced by the Waviness widget [equation (3) (Sanchez del Rio & Marcelli, 1992 ▸)]. Figs. 5 ▸(*a*)–5(*c*) show three combinations of 0th, 1st, 20th and 40th harmonics with the same *C*_*n*_ values [equation (4)]. Their corresponding residual wavefront and residual wavefront derivative are plotted in Figs. 5 ▸(*d*) and 5 ▸(*e*), respectively,

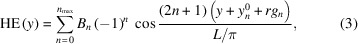

where

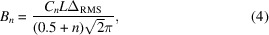

where *L*, Δ_RMS_, *r*, *y* and *n*_max_ are, respectively, the mirror length, the RMS slope error, a random number (0 < *r* < 1), the position along the tangential direction, and the maximum required harmonic. *C*_*n*_, 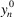 and *g*_*n*_ are, respectively, weighting coefficients, initial shift values and a fraction of *L* (∼0.1*L*) for the *n*th harmonic (Sanchez del Rio & Marcelli, 1992 ▸). These parameters are adjusted manually to obtain the best simulated height error profile compared with the measured one.

The harmonic involved in the mirror height error can be calculated inversely by expanding either the residual wavefront error or residual wavefront derivative as shown in Fig. 5 ▸. However, the size of the higher-frequency oscillations is larger in the wavefront derivative compared with the wavefront error. Comparing the height error with the residual wavefront derivative highlights the advantages of the wavefront derivative as a method to recalculate the height error profile. The wavefront error is also sensitive to any other height error introduced due to clamping or mechanical stress in the working condition. Therefore, this method can be proposed as *in situ* metrology for a mirror in good comparison with other *in situ* metrology techniques that have been developed in recent years (Mercère *et al.*, 2003 ▸; Rutishauser *et al.*, 2011 ▸; Yumoto *et al.*, 2006 ▸). Choosing an appropriate knife-edge step (about 1/100th to 1/50th of the focal size), detector pixel size and detector to focal distance are essential to determine an accurate height error profile. The knife-edge step provides the height error accuracy while the pixel size and detector distances are determined from the focused beam divergence and the required spatial resolution along the mirror length. For example, for 200 nm focal size and 0.235 m focal distance, a 1 nm knife step, 3 µm detector pixel size and 0.5 m focal to detector distance are required to obtain an error profile containing harmonics up to *n* = 20.

The contraction in both amplitude and period by going to the higher aperture (closer to the focus) needs to be considered for the reverse analysis. The contraction varies by the ratio of the focal distance to mirror length and becomes dominant for highly curved mirrors. A program was written to reconstruct the mirror height profile from the calculated residual wavefront derivative which is in good agreement. This height error is then used toward designing a generic or customized wavefront corrector which is discussed in the following section.

### 3.2. Wavefront correction

Residual wavefront errors have unwanted effects on the in-focus and out-of-focus beam profile including beam broadening, peak intensity and inhomogeneous structures. Thus, wavefront correction is essential to maintain a good focal profile quality. It has been shown that a refractive structure, either adaptive (Laundy *et al.*, 2019 ▸) or customized (Sawhney *et al.*, 2016 ▸), inserted into the beam path can correct the wavefront error. An adaptive refractor has the advantage of dynamic correction over a wide energy range and the possibility of correction of wavefront errors introduced by the upstream optical elements such as crystal monochromator or prefocusing mirrors. Correcting from low to high spatial frequencies may require a few inline correctors.

Here, we have developed a Python program to design a suitable adaptive or customized refractive corrector. This is done by simulating the deflection of an incident beam at the surface of the refractor in order to cancel out the wavefront error caused by the optical element imperfections. The inputs to this program are the working photon energy, the refractive index of the structure material and the residual wavefront error profile. The wavefront correction is proportional to the real part decrement of the refractor material at the X-ray beam energy multiplied by the X-ray pathlength in the refractor, which makes it easier to extend the calculated profile to a wider range of X-ray energies. A series of customized structures fabricated on a single chip layout can therefore be used to correct the wavefront over a wide energy range.

**Figure 6:**
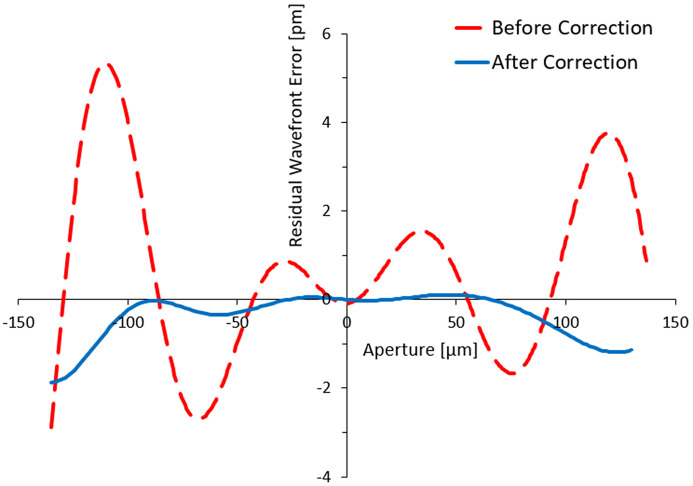
Residual wavefront error before (dashed line) and after (solid line) wavefront correction.

Several ray-tracing and propagation simulations were carried out to investigate the applicability of this correction. Fig. 6 ▸ shows the residual wavefront error calculated by the knife-edge method for a few harmonics height error profiles on the VFM mirror before and after the correction. Ideally, all the wavefront error harmonics can be corrected by this method to find a zero-wavefront error. However, a part of the wavefront error amplitude remains due to the approximations used in this program. This has a small contribution to the RMS values (degree of correction or figure of merit) of the corrected wavefront, reducing from 2.1 pm to 0.6 pm in this case.

**Figure 7:**
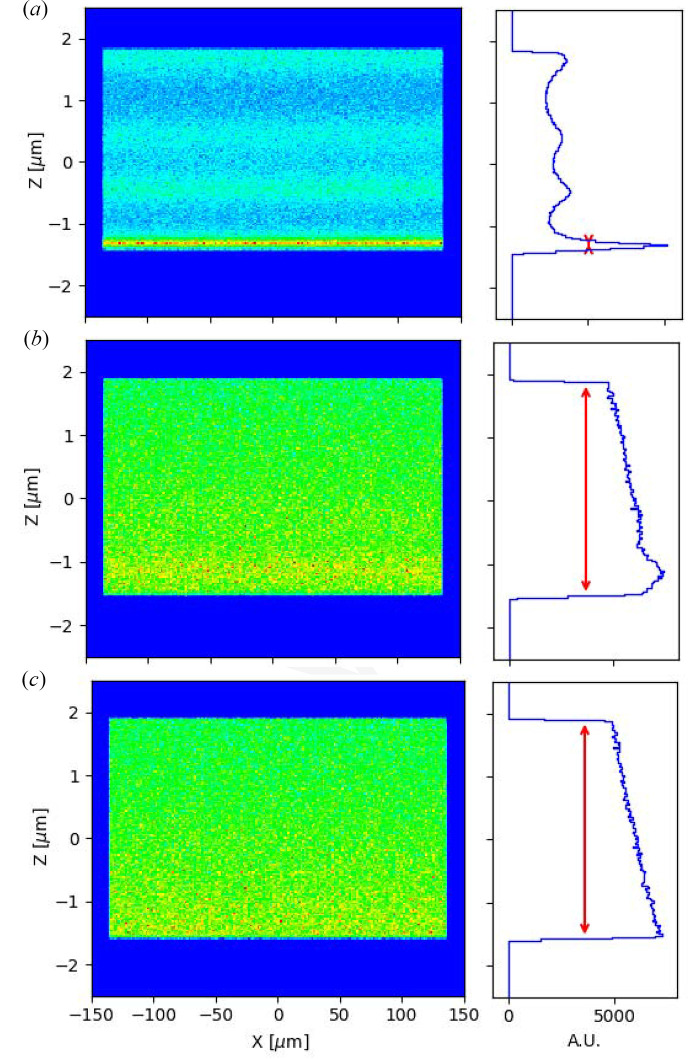
Beam profile 3 mm downstream of the focus (*a*) before wavefront correction, (*b*) after correction and (*c*) compared with an ideal focusing mirror.

The refractive corrector compensates for the height error profile of the mirror that is the main source of the beam inhomogeneous structure at the out-of-focus position (Cocco *et al.*, 2022 ▸). Fig. 7 ▸ shows the beam profile at 3 mm downstream of the focal plane for the height error (shown in Fig. 6 ▸) before and after correction. A round source of size 10 µm is considered for the out-of-focus calculation. The beam profile before correction [Fig. 7 ▸(*a*)] shows an inhomogeneous beam profile including four peaks distributed in the whole focal spot. This is changed to an almost uniform beam intensity distribution after wavefront correction [Fig. 7 ▸(*b*)]. This is very similar to the out-of-focus beam distribution that is produced by an ideal mirror where there are no high or slope errors [Fig. 7 ▸(*c*)]. This correction has many applications in beamlines that use a variable beam size such as macromolecular crystallography beamlines.

**Figure 8:**
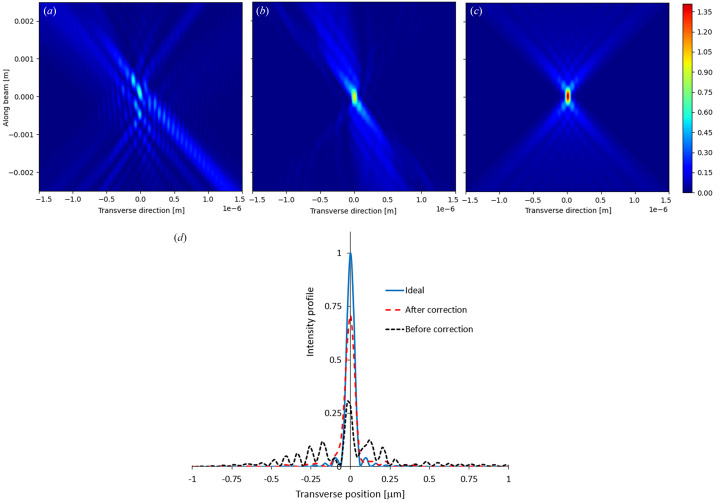
Wavefront propagation along the longitudinal position at the diffraction limit condition before wavefront correction (*a*) and after the correction (*b*) compared with an ideal focusing mirror (*c*). The colour bar shows the beam intensity in arbitrary units. The transverse beam profiles at the ideal image plane for the three cases and their FWHM values are also compared (*d*).

Fig. 8 ▸ shows the wave propagation of a coherent beam from an aperture at the optical element to its focus position. It has been calculated for a more pronounced wavefront error, *i.e.* horizontal focusing mirror of the Test beamline at Diamond Light Source (Laundy *et al.*, 2019 ▸). The vertical axis is along the beam direction over a range of ±2.5 mm around the focal position, while the horizontal axis shows the transverse direction. The diffraction-limited size is about 100 nm. Fig. 8 ▸(*a*) shows the uncorrected propagation and, as can be seen, there is significant intensity separated from the main focus peak. Fig. 8 ▸(*b*) shows the profile after the correction and Fig. 8 ▸(*c*) shows the profile for an ideal wavefront. Fig. 8 ▸(*b*) shows significant improvement in both the focal spot profile and the intensity of the main peak compared with Fig. 8 ▸(*a*). The normalized beam intensity at the image plane for the three cases is plotted in Fig. 8 ▸(*d*). The peak intensity is reached at about 70% of its ideal intensity after correction and the focal size approaches the ideal diffraction limit of 100 nm. These results corroborate well with our previous results published elsewhere (Laundy *et al.*, 2019 ▸).

## 4. Summary and conclusion

We have developed and demonstrated a knife-edge simulation tool to analyse the wavefront of an elliptical mirror using the *SHADOW* ray-tracing code. The simulated wavefront derivative is then used to reconstruct the mirror height error and provide accurate *in situ* metrology of the focusing mirror. The developed code provides a quick way of designing adaptive or customized refractive structures to correct the X-ray wavefront of a mirror by minimizing the mirror’s residual wavefront error. Further, wavefront correction has helped in improving the beam intensity not only at the focus but also in the structureless beam distribution at and out of focus. The simulation results were compared with measured data on the Test beamline at Diamond Light Source (Laundy *et al.*, 2019 ▸; Dhamgaye *et al.*, 2023 ▸) and the outcome provides significant confidence in the application of this method which will reduce future experiments’ time and effort. Involving the diffraction effects in the *SHADOW* ray-tracing code suggests our method is an alternative for beamline design of the fourth-generation light sources.

## 5. Data availability

The authors are extending the wavefront analysis program to other optics and making it more user-friendly. Therefore, both data and code are available from the corresponding author upon reasonable request.
